# Twist2 contributes to cisplatin-resistance of ovarian cancer through the AKT/GSK-3β signaling pathway

**DOI:** 10.3892/ol.2014.1816

**Published:** 2014-01-21

**Authors:** TIAN WANG, YAN LI, ABIDAN TUERHANJIANG, WENWEN WANG, ZHANGYING WU, MING YUAN, MAYINUER MAITITUOHETI, SHIXUAN WANG

**Affiliations:** Department of Obstetrics and Gynecology, Tongji Hospital, Tongji Medical College, Huazhong University of Science and Technology, Wuhan, Hubei 430030, P.R. China

**Keywords:** Twist2, cisplatin, chemoresistance, AKT/GSK-3β pathway, ovarian cancer

## Abstract

Cisplatin is regularly used in the treatment of ovarian cancer. However, the drug only provides a modest survival advantage, primarily due to chemoresistance and the upregulation of antiapoptotic machineries in ovarian cancer cells. Therefore, targeting the mechanisms responsible for cisplatin resistance in ovarian cancer cells may improve the therapeutic outcomes. Twist basic helix-loop-helix transcription factor 2 (Twist2) is a novel zinc finger transcription factor that has been indicated to be an important inducer of epithelial-mesenchymal transition, which has been shown to be involved in various phases of tumorigenicity and progression. However, whether Twist2 suppression increases the chemosensitivity of ovarian cancer cells to chemotherapeutic agents remains unclear. In the present study, Twist2 expression was found to differ between human ovarian cisplatin-sensitive cancer cell line, OV2008, and the resistant variant, C13K cells. Twist2 plasmids or RNA interference were then utilized to alter Twist2 expression in OV2008 or C13K cells, respectively, to further assess apoptosis, cell viability and cell growth, as well as a possible mechanism. The results of the present study indicated that Twist2 plays a crucial role in the chemoresistance of ovarian cancer. In addition, the downregulation of Twist2 expression may facilitate apoptosis and recover the sensitivity of chemoresistant ovarian cancer through the protein kinase B/glycogen synthase kinase-3β pathway. Therefore, Twist2 depletion may be a promising approach to ovarian cancer therapy.

## Introduction

Ovarian cancer is the fifth most common cause of cancer-related mortality in females and accounts for the highest tumor-associated mortality of gynecological malignancies (1). Late diagnosis of ovarian cancer and ineffective chemotherapy has resulted in the number of mortalities from ovarian cancer exceeding that of any other cancer of the female reproductive system (2). More than 70% of patients with ovarian cancer are diagnosed in the advanced stages (International Federation of Gynecology and Obstetrics stages III and IV), where the cancer has spread beyond the ovary and treatment becomes increasingly ineffective (3). Currently, the preferred treatment of human ovarian cancer is cisplatin-centered chemotherapy, which can markedly decrease the mortality rate and lengthen the survival time for patients. However, a major obstacle in ovarian cancer treatment is the development of drug resistance. The mechanism responsible for cisplatin chemoresistance in ovarian cancer remains poorly understood (4).

Recently, twist basic helix-loop-helix transcription factor 2 (Twist2), a highly homologous protein of Twist1, has attracted increasing attention. Overexpression of Twist2 has been reported to correlate with poor prognosis of colorectal cancer, as well as head and neck squamous cell carcinomas (5,6). Zhou *et al* (7) and Li *et al* (8) indicated that Twist2 is associated with the invasion and metastasis of salivary adenoid cystic carcinoma, cervical malignant conversion and tumor metastasis separately. In addition, Koh *et al* (9) reported that Twist2 increased the resistance to galectin-1-mediated apoptosis, which facilitated the progression of T cells into tumors. Twist2 is also considered to be an inducer of epithelial-mesenchymal transition (EMT) (10), a well-known process involved in embryogenesis (11), tumor invasion, metastasis (12) and drug resistance (13). Evidently, Twist2 plays a critical role in human tumors. Thus, the present study investigated Twist2 expression patterns in ovarian cancer, the role in chemoresistance and also a possible underlying mechanism.

The aim of the present study was to investigate the Twist2 expression pattern in a cisplatin-sensitive ovarian cancer cell line (OV2008) and the resistant variant (C13K), and to determine the effect Twist2 has on the regulation of cell growth and cisplatin-induced apoptosis in ovarian cancer cells. Furthermore, the effect Twist2 has on the protein kinase B (AKT)/glycogen synthase kinase (GSK)-3β signaling pathway was investigated.

## Materials and methods

### Cell lines and culture

A cisplatin-sensitive ovarian cancer cell line (OV2008) and the resistant variant (C13K) were supplied by Dr Rakesh Goel from the Ottawa Regional Cancer Center (Ottawa, ON, Canada). Cells were maintained in RPMI-1640 complete medium supplemented with 2 mM glutamine and 10% fetal bovine serum (FBS) at 37°C in a humidified atmosphere containing 5% CO_2_.

### Chemicals and antibodies

RPMI-1640, FBS, Lipofectamine 2000 and TRIzol reagent were purchased from Invitrogen Life Technologies (Carlsbad, CA, USA). Cisplatin was obtained from QiLu Pharmaceutical Co., Ltd. (Jinan, China) and the phosphoinositide 3-kinase inhibitor, LY294002, methyltetrazolium (MTT) and dimethyl sulfoxide (DMSO) were obtained from Sigma-Aldrich (St. Louis, MO, USA). pcDNA3.1(+) (vector), pcDNA3.1(+)/Twist2 (Twist2), Twist2 siRNA (si-Twist2) and negative control siRNA (si-NC) were purchased from Guangzhou RiboBio, Co., Ltd. (Guangzhou, China). Primary antibody against Twist2 was obtained from Abcam, Inc. (Burlingame, CA, USA), while those against AKT, AKT Ser-473, GSK-3β and GSK-3β Ser-9 were obtained from Cell Signaling Technology, Inc. (Beverley, MA, USA). Polymerase chain reaction (PCR) primers were purchased from Invitrogen Life Technologies.

### Cell transfection

For transient transfection, cells were seeded in six-well plates at a density of 5×10^4^ cells/well and incubated at 37°C in an atmosphere with 5% CO_2_ for 12 h. When the cells were 30–50% confluent, they were transfected using Lipofectamine 2000 transfection reagent, according to the manufacturer’s instructions.

### Cell viability assay and cellular growth rate

Cell viability and IC_50_ values (drug concentration causing 50% inhibition of cell growth) were analyzed with the MTT assay. For transient transfection, cells were seeded at 5×10^3^ cells/well in 96-well plates one day prior to transfection. The MTT assay was performed prior to transfection and then at 24, 48, 72 and 96 h following transfection. For the assay, 20 μl MTT (5 mg/ml) was added to each well and the cells were incubated for 4 h prior to the addition of 180 μl DMSO for 20 min. The absorbance of the wells was then measured with a microplate reader at a test wavelength of 570 nm and a reference wavelength of 630 nm. Appropriate controls lacking cells were included to determine the background absorbance. The response to drug treatment was assessed by standardizing the treatment groups to the untreated control. Cellular growth curves were plotted using the cellular viability values assessed by the MTS, a colorimetric method used to determine the number of viable cells using [3-(4,5-dimethyl-2-yl)-5-(3-carboxymethoxyphenyl)-2(4-sulfophenyl)-2H-tetrazolium, inner salt; MTS], according to the manufacturer’s instructions (Promega, Madison, WI, USA).

### Quantitative PCR (qPCR)

Total RNA was extracted from cultured cells using TRIzol reagent. For each sample, 2 μg RNA was reverse transcribed using a ReverTra Ace qPCR kit (Toyobo, Co., Ltd., Osaka, Japan), according to the manufacturer’s instructions. qPCR was performed using SYBR Green qPCR Master Mix (DBI, Inc., Hazleton, PA, USA) on a CFX Connect real-time system (Bio-Rad Laboratories, Inc., Berkeley, CA, USA). The conditions were as follows: 40 cycles of three-step PCR (95°C for 40 sec, 60°C for 50 sec and 72°C for 30 sec) following initial denaturation (95°C for 5 min). All primers were supplied by Invitrogen Life Technologies. Primer sequences were as follows: Forward, 5′-GAGCGACGAGATGGACAATAAGA-3′ and reverse, 5′-ATGCGCCACACGGAGAA-3′ for Twist2 (product size, 84 bp); forward, 5′-TGCACCACCAACTGCTTAGC-3′ and reverse 5′-GGCATGGACTGTGGTCATGAG-3′ for GAPDH (housekeeping gene; product size, 87 bp).

### Western blotting

Following transfection, cells were lysed in ice-cold radioimmunoprecipitation assay lysis buffer containing a protease inhibitor cocktail. A total of 60 μg protein was separated by 10% SDS-PAGE and transferred to polyvinylidene fluoride membranes. Following blocking with Tris-buffered saline containing 5% skimmed milk at room temperature for 1 h, the membranes were incubated with primary antibody at 4°C for 12 h. The membranes were then incubated with horseradish peroxidase-conjugated anti-mouse/rabbit antibodies at a dilution of 1:3,000 at room temperature for 1 h. Signals were detected on X-ray film using an enhanced chemiluminescent detection system (Pierce Biotechnology, Inc., Rockford, IL, USA). Loading differences were normalized against a monoclonal GAPDH antibody.

### Flow cytometry

All samples were washed in phosphate-buffered saline and resuspended in 200 μl binding buffer. Next, 5 μl Annexin-V-fluorescein isothiocyanate and 10 μl propidium iodide (PI; 1 μg/ml) were added and the cell suspension was incubated in a dark chamber at room temperature for 1 h. Cell-cycle profiles were then determined using a FACSCalibur flow cytometer (BD Biosciences, Franklin Lakes, NJ, USA) and data were analyzed using CellQuest software (BD Biosciences).

### Statistical analysis

All experiments were repeated at least three times and the data are expressed as the mean ± standard deviation. P<0.05 was considered to indicate a statistically significant difference. Statistical analysis was conducted with SPSS 18.0 for Windows (SPSS, Inc., Chicago, IL, USA) using the Student’s t-test.

## Results

### Chemoresistance increases in ovarian cancer due to high expression levels of Twist2

**Figure 1:**
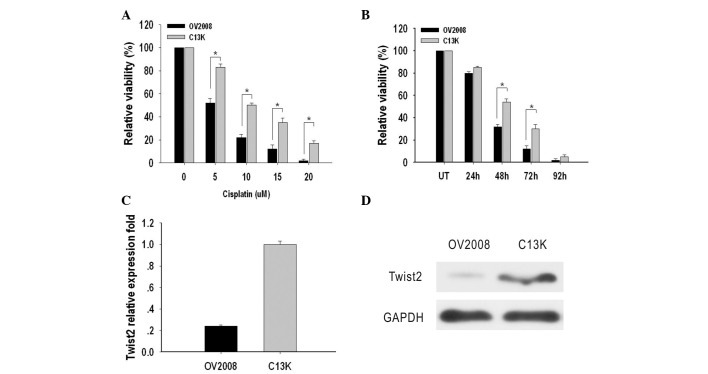
Expression of Twist2 and cisplatin-induced cytotoxicity and apoptosis in OV2008 and C13K cells. (A) The cytotoxic effect of cisplatin on OV2008 and C13K cells was determined by an MTT assay. (B) Cell viability was assessed with MTT assay and expressed as a percentage relative to the respective untreated controls at the same point. OV2008 and C13K cells were untreated or treated with 10 μM cisplatin for various durations.^*^P<0.05. (C) Twist2 mRNA expression was detected with quantitative polymerase chain reaction. Glyceraldehyde 3-phosphate dehydrogenase (GAPDH) was co-amplified as the internal control. (D) Expression of Twist2 protein was detected using western blot analysis in total cell extracts. GAPDH was reprobed to confirm equal protein loading.

The cytotoxic effect of cisplatin on OV2008 and C13K cells was determined by an MTT assay. IC_50_ values were used to indicate the levels of cytotoxicity. The IC_50_ values in OV2008 and C13K cells were 5±0.3 μM and 10.0±1.2 μM, respectively, which indicated that the OV2008 cells were more sensitive to cisplatin-induced cytotoxicity compared with C13K cells (Fig. 1A). Next, OV2008 and C13K cells were treated with 10 μM cisplatin for 24, 48, 72 and 96 h in order to measure cell viability. The results indicated that C13K cells were significantly more viable after 48 h (1.6 fold) and 72 h (2.5 fold) when compared with OV2008 cells. After 96 h, cell death had occurred in ~95% of the cells in the two cell types (Fig. 1B).

Expression levels of Twist2 in these two cell lines were compared by qPCR and western blotting. The mRNA expression level of Twist2 in OV2008 and C13K cells was detectable and relative absorbance values were 0.24±0.012 and 1.00±0.03, respectively. Protein expression of Twist2 in OV2008 and C13K cells was assayed and relative absorbance values were 0.15±0.01 and 1.02±0.02, respectively. The mRNA and protein expression levels of Twist2 in C13K cells were significantly higher compared with those in OV2008 cells (P<0.05; Fig. 1C and D).

### Twist2 confers resistance to cisplatin in ovarian cancer cells

**Figure 2:**
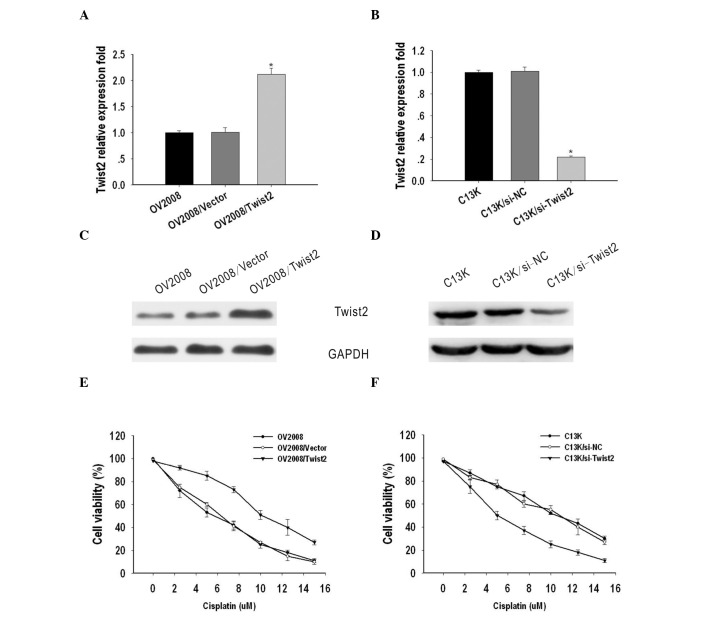
Twist2 confers resistance to cisplatin in ovarian cancer cells. (A) Validating the efficiency of pcDNA3.1(+)/Twist2 in OV2008 cells by quantitative polymerase chain reaction (qPCR). PcDNA3.1(+)/Twist2 significantly increased Twist2 at mRNA levels. (B) Validating the efficiency of si-Twist2 in C13K cells by qPCR. si-Twist2 significantly ablated endogenous Twist2 at the mRNA level. ^*^P<0.05. (C) Validating the efficiency of pcDNA3.1(+)/Twist2 in OV2008 cells by western blotting. PcDNA3.1(+)/Twist2 significantly upregulated Twist2 at the protein level. (D) The efficiency of si-Twist2 in C13K cells was validated by western blotting. si-Twist2 significantly ablated endogenous Twist2 at the protein level. (E) IC_50_ level of OV2008/Twist2 induced by cisplatin was markedly higher than those of the OV2008 and OV2008/Vector cells. (F) IC_50_ level of C13K/si-Twist2 induced by cisplatin was markedly lower than those of C13K and C13K/si-NC cells.

To investigate the possible role of Twist2 on the sensitivity of ovarian cancer to cisplatin, OV2008 and C13K cells were transiently transfected with vector/Twist2 or si-NC/si-Twist2, respectively. Total RNA and protein were isolated and analyzed by qPCR and western blotting 48 h after transfection. Compared with the OV2008 and OV2008/vector cells, the expression of Twist2 was markedly upregulated in OV2008/Twist2 cells at the mRNA and protein level (Fig. 2A and C). By contrast, Twist2 expression was suppressed in C13K cells transfected with si-Twist2, when compared with C13K and C13K/si-NC cells (Fig. 2B and D).

To evaluate the biological significance of Twist2 on cell sensitivity to cisplatin, an MTT assay was performed. The IC_50_ values of cisplatin for OV2008, OV2008/vector and OV2008/Twist2 cells were 5±0.12, 6±0.281 and 10±0.193 μM, respectively, while the IC_50_ values for C13K, C13K/si-NC and C13K/si-Twist2 cells were 10.21±0.12, 10.3±0.281 and 5.4±0.193 μM, respectively. These results indicate that Twist2 contributes to cisplatin-resistance in ovarian cancer cells (Fig. 2E and F).

### Twist2 regulates cisplatin-induced apoptosis and cell growth in ovarian cancer

**Figure 3:**
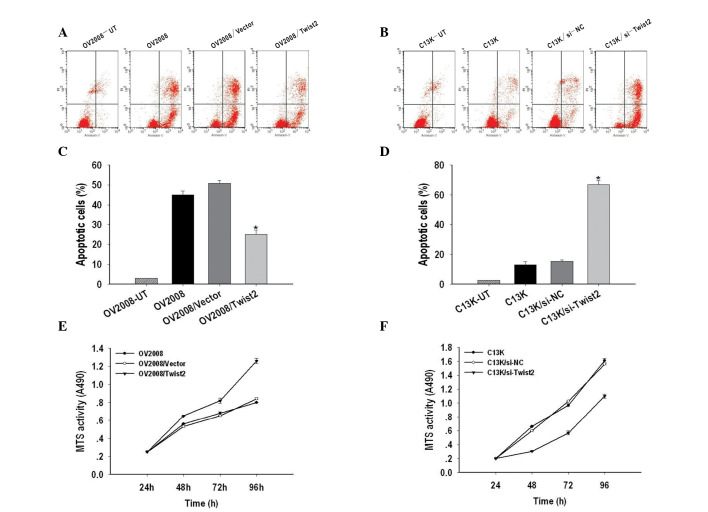
Twist2 regulates cisplatin-induced apoptosis and cell growth in ovarian cancer. (A) Representative FACS analyses for the induction of apoptosis in Twist2 on OV2008, OV2008/Vector and OV2008/Twist2 cells were harvested at 48 h and stained with Annexin-V-fluorescein isothiocyanate (FITC) and propidium iodide (PI) followed by FACScan flow cytometric analysis. (B) Representative FACS analyses for apoptosis induction of C13K, C13K/si-NC and C13K/si-Twist2 cells were harvested at 48 h and stained with Annexin-V-FITC and PI followed by FACScan flow cytometric analysis. (C) Quantitative analysis of the population of total apoptotic cells. The apoptotic ratio of OV2008/Twist2 was significantly lower than those of OV2008 and OV2008/Vector cells. (D) The apoptotic ratio of C13K/si-Twist2 was remarkably higher than those of C13K and C13K/si-NC cells. Three independent experiments were conducted and the data shown are the means ± SEM. (E) The 3-(4,5-dimethyl-2-yl)-5-(3-carboxymethoxyphenyl)-2(4-sulfophenyl)-2H-tetrazolium, inner salt] MTS assay was performed in order to assess the growth of OV2008, OV2008/Vector and OV2008/Twist2 cells at 24, 48, 72 and 96 h, revealing a significant increase in the proliferation rate for OV2008/Twist2 cells. (F) The MTS assay was performed to assess the growth of C13K, C13K/si-NC and C13K/si-Twist2 cells at 24, 48, 72 and 96 h, showing a significant decrease of proliferation rate for C13K/si-Twist2 cells. The relative ratio of cell proliferation to untransfected cells was measured and the data shown are the means ± SEM of three independent experiments.

The effects of apoptosis were examined in Twist2 silenced ovarian cancer cells using Annexin-V and PI double-staining and measured by flow cytometry. As shown in Fig. 3A and C, the apoptotic rate of OV2008/Twist2 (21.7%) was significantly lower than OV2008 (56.95%) and OV2008/vector (60.7%) cells. In addition, Twist2 silencing resulted in 81.01% of C13K cells being apoptotic, while the apoptotic rate of C13K and C13K/si-NC cells was 15 and 18%, respectively (P<0.05) (Fig. 3B and D).

The role of Twist2 in the growth of ovarian cancer cells was determined by an MTS assay. As shown in Fig. 3E and F, Twist2 upregulation promoted cell growth of OV2008 cells, while downregulation of Twist2 by transient transfection of si-Twist2 in C13K cells caused significant inhibition of cell proliferation. These results indicate that Twist2 regulates cisplatin-induced apoptosis and cell growth in ovarian cancer.

### Twist2 mediates cisplatin resistance and apoptosis via regulating the AKT/GSK-3β pathway

**Figure 4:**
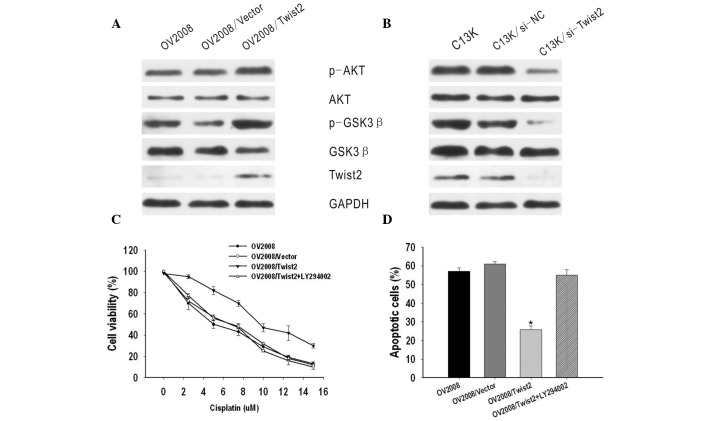
Twist2 mediates cisplatin resistance and apoptosis accompanied with the activation of AKT/GSK-3β pathway. (A) Cell lysates were subjected to SDS-PAGE followed by western blotting using specific antibodies against Twist2 and Akt/GSK3β pathway proteins. P-Akt and p-GSK3β were significantly up-regulated by Twist2 in OV2008. (B) P-Akt and p-GSK3β proteins were remarkably down-regulated by si-Twist2 in C13K. (C) Effect of cisplatin on cell viability in OV2008, 0V2008/Vector, OV2008/Twist2 and OV2008/Twist2+LY294002. OV2008/Twist2+LY294002 demonstrated a diminished viability in response to cisplatin, compared with OV2008/Twist2. (D) Apoptotic rate was evaluated in order to determine whether the Akt/GSK3β pathway is involved in the synergistic effect of Twist2. OV2008/Twist2+LY294002 demonstrated an increased apoptosis in response to cisplatin, compared with OV2008/Twist2. Results are expressed as the means ± SEM of three independent experiments. ^*^P<0.05.

A previous study revealed that AKT/GSK-3β pathway activation plays an important role in cisplatin resistance of breast cancer (14). Thus, the present study investigated the correlation between the AKT/GSK-3β pathway and Twist2 expression in ovarian cancer. It was identified that phosphorylated AKT and GSK-3β expression, but not total AKT or GSK-3β expression, were markedly increased by the upregulation of Twist2 in OV2008 cells, but decreased in C13K/si-Twist2 cells, as compared with C13K and C13K/si-NC cells (Fig. 4A and B).

To further investigate the molecular mechanisms, an inhibitor of PI3K/AKT (LY294002) was used. GSK-3β is the kinase located downstream of the PI3K/AKT pathway. It was identified that OV2008/Twist2 cells treated with LY294002 appeared to be more sensitive to cisplatin than OV2008/Twist2 cells not treated with the inhibitor. The IC_50_ value of OV2008/Twist2 + LY294002 was markedly lower compared with that of OV2008/Twist2 cells (P<0.05) and similar to that of OV2008 and OV2008/vector cells (P>0.05) (Fig. 4C). Subsequent cisplatin treatment markedly enhanced apoptotic cell death. In addition, the apoptotic ratio of OV2008/Twist2 + LY294002 was 54.92±3.01%, which was significantly higher compared with that of OV2008/Twist2 (25.73±2.2%) (P<0.05) and similar to that of OV2008 (57.07±1.92%) and OV2008/vector (60.95±1.34%) cells (P>0.05) (Fig. 4D). These results indicate that the AKT/GSK-3β signaling pathway may be involved in Twist2-induced cisplatin-resistance.

## Discussion

Ovarian cancer is the leading cause of mortality among gynecological cancers. A major cause of the high mortality rates in ovarian cancer is chemotherapy resistance, particularly cisplatin resistance (15). Cancer cells develop resistance to chemotherapy by inactivating apoptotic factors and enhancing survival pathways that antagonize apoptosis signals (16). The balance between survival and apoptotic signals determines the sensitivity of cells to chemotherapy. However, in ovarian cancer, the molecular mechanisms leading to cisplatin chemoresistance remain poorly understood.

Twist2 was first identified in 1995 and has been shown to share high homology and overlapping expression patterns with Twist1 (8,17). Extensive studies in previous years have focused on Twist1 and have identified correlations between the development of acquired metastatic ability, stem cell-like characteristics and chemoresistance in various human cancers (18–21). Although gene deletion experiments have shown that Twist1 and Twist2 have specific functional similarity and redundancy (22,23), Tukel *et al* demonstrated that these two genes exhibit non-redundant functions in skin and bone development, highlighting the importance of studying Twist1 and Twist2 as separate entities (24). Similarly to Twist1, Twist2 has previously been reported to be implicated in cell lineage determination and differentiation (25). Upregulation of Twist2 expression has been detected in a wide range of human cancers and Twist2 has already been shown to be a significant molecule in specific solid tumors (6). However, the role that Twist2 plays in drug resistance and the possible underlying mechanism in ovarian cancer has not yet been established.

In the present study, a pair of chemosensitive (OV2008) and chemoresistant (C13K) ovarian cancer cell lines were used to investigate the possible roles of Twist2 in the regulation of cisplatin-mediated apoptosis and cisplatin resistance in human ovarian epithelial cancer. C13K cells were found to be more resistant to cisplatin-induced cytotoxicity than OV2008 cells. In addition, Twist2 expression in the OV2008 cell line was significantly lower compared with that in the C13K cell line. These results indicate that the loss of Twist2 may be involved in cisplatin sensitivity of ovarian cancer cells and may play an important role in the chemoresistance of ovarian cancer cells.

However, the mechanism by which Twist2 contributes to chemoresistance remains unclear. The fate of cancer cells in response to a chemotherapeutic agent is a consequence of the overall apoptotic capacity of the cell (26). Successful transfections of OV2008 cells with Twist2 and C13K cells with si-Twist2 were performed with Lipofectamine 2000. It was found that upregulation of Twist2 expression significantly decreased cisplatin-induced apoptosis and promoted cell growth. By contrast, downregulation of Twist2 expression was found to be an effective method of reversing the resistance of C13K cells to cisplatin and sensitizing the cells to cisplatin-induced apoptosis. Therefore, the results indicate that combination chemotherapy targeting Twist2 expression is likely to improve the treatment of ovarian cancer.

The PI3K/AKT antiapoptotic and survival pathway has been reported to play a crucial role in cisplatin resistance (27). The activation of AKT requires phosphorylation at the Ser-473 and Thr-308 sites; thus, the level of phosphorylated-AKT (Ser-473) represents the activity of AKT (28). Phosphorylated AKT promotes survival by phosphorylating and inactivating proapoptotic factors, including GSK-3β (29). A number of studies have shown that the activated AKT/GSK-3β pathway participates in the EMT process and contributes to the aggressive phenotype and chemoresistance in several human cancers (30–32). Thus, in the present study, the AKT/GSK-3β pathway was investigated to determine whether it played a role in Twist2-mediated cisplatin-resistance in ovarian cancer. The results showed that the phosphorylation of AKT at Ser-473 and GSK-3β at Ser-9 in OV2008/Twist2 cells was markedly higher than that in OV2008 and OV2008/vector cells. However, there was no difference in total AKT and GSK-3β protein expression. In addition, LY294002, an inhibitor of PI3K/AKT, effectively reversed Twist2-induced cisplatin-resistance. Thus, targeting this signaling pathway may be an effective approach to treating cisplatin-resistance in ovarian cancer.

In conclusion, the present study demonstrates that Twist2 plays a crucial role in the chemoresistance of ovarian cancer. Downregulation of Twist2 expression facilitated apoptosis and recovered the sensitivity of chemoresistant ovarian cancer through the AKT/GSK-3β pathway. However, further clarification of functional characterization is required. The results of the current study provide support for this potential novel gene therapy with Twist2 for the treatment of chemoresistant human ovarian cancer.
